# Detection and characterization of Langya virus in *Crocidura lasiura* (the Ussuri white-toothed shrew), Republic of Korea

**DOI:** 10.1016/j.onehlt.2025.101017

**Published:** 2025-03-19

**Authors:** Augustine Natasha, Sarah E. Pye, Kyungmin Park, Shivani Rajoriya, Intae Yang, Jieun Park, Haryo Seno Pangestu, Jongwoo Kim, Yeonsu Oh, Carolina B. López, Jin-Won Song, Won-Keun Kim

**Affiliations:** a Department of Microbiology, College of Medicine, Hallym University, Chuncheon 24252, Republic of Korea; b Department of Molecular Microbiology and Center for Women's Infectious Disease Research, Washington University School of Medicine in St. Louis, MO, United States of America.; c Department of Microbiology, Korea University College of Medicine, Seoul 02841, Republic of Korea; d Institute for Viral Diseases, Korea University College of Medicine, Seoul 02841, Republic of Korea; e BK21 Graduate Program, Biomedical Sciences, Korea University College of Medicine, Seoul 02841, Republic of Korea; f College of Veterinary Medicine and Institute of Veterinary Science, Kangwon National University, Chuncheon 24341, Republic of Korea; g Institute of Medical Science, College of Medicine, Hallym University, Chuncheon 24252, Republic of Korea

**Keywords:** *Parahenipavirus*, Langya virus, *Crocidura lasiura*

## Abstract

Langya virus (LayV) is the only documented zoonotic agent within the shrew borne *Parahenipavirus* genus. Other *Parahenipavirus* species, including Gamak virus and Daeryeong virus, have been discovered in the Republic of Korea, highlighting the prevalence of this genus in the region. We retrospectively analyzed metagenomic next-generation sequencing of two *Crocidura lasiura* (the Ussuri white-toothed shrew) kidney samples from 2017, followed by paramyxovirus screening of 24 kidney samples from the same species collected in 2023. The LayV positivity rate was 12.5 % (3 of 24). Amplicon-based sequencing was subsequently developed to obtain the complete viral sequences. Five complete genomes of Langya virus Korea (LayV KOR) were identified: two from 2017 samples and three from 2023 samples. LayV KOR exhibited approximately 80 % and 95.5 % homology at the nucleotide and amino acid levels, respectively. Phylogenetic analysis underscored the close relationship between LayV KOR and LayV from China. This study represents the first detection of LayV complete sequences in shrews outside of China.

## 1 Introduction

The *Parahenipavirus* is a recently established genus in the *Paramyxoviridae* family, comprising viruses previously referred to as henipa-like viruses [1]. The only shrew-borne zoonotic *Parahenipavirus* identified to date is the *Parahenipavirus langyaense* (Langya virus; LayV), first discovered in 2018 from patients with fever in China [2]. Previous studies on the attachment glycoprotein and fusion protein showed distinct structures and antigenic potential compared to zoonotic henipaviruses, indicating the differences between *Parahenipavirus* and its closely related genus [3,4]. The shrew species *Crocidura lasiura* and *C. shantungensis* are found to be natural hosts of LayV. *Parahenipavirus gamakense* (Gamak virus; GAKV) from *C. lasiura* and *Parahenipavirus daeryongense* (Daeryeong virus; DARV) were found from *C. shantungensis* in the Republic of Korea (ROK), respectively [5]. These findings suggest the presence and potential prevalence of *Parahenipavirus* species in the ROK. Despite the wide distribution of *Crocidura* species, there have been no reports of LayV outside of China to date.

In this study, we delineated the complete genome sequence of LayV identified from five *C. lasiura* during surveillance monitoring in the ROK, using a combination of metagenomic and amplicon-based sequencing. Through retrospective analysis of prior sequencing data, we identified LayV sequences from Korean *C. lasiura* samples as early as 2017. Analysis of these genome sequences revealed the presence of a previously unreported hypothetical protein domain between the matrix and fusion proteins. Phylogenetic analysis demonstrated a close evolutionary relationship between LayVs from the ROK and China. This is the first report of LayV outside China, highlighting the cross-border circulation of emerging *Parahenipavirus*.

## 2 Materials and method

**Fig. 1:**
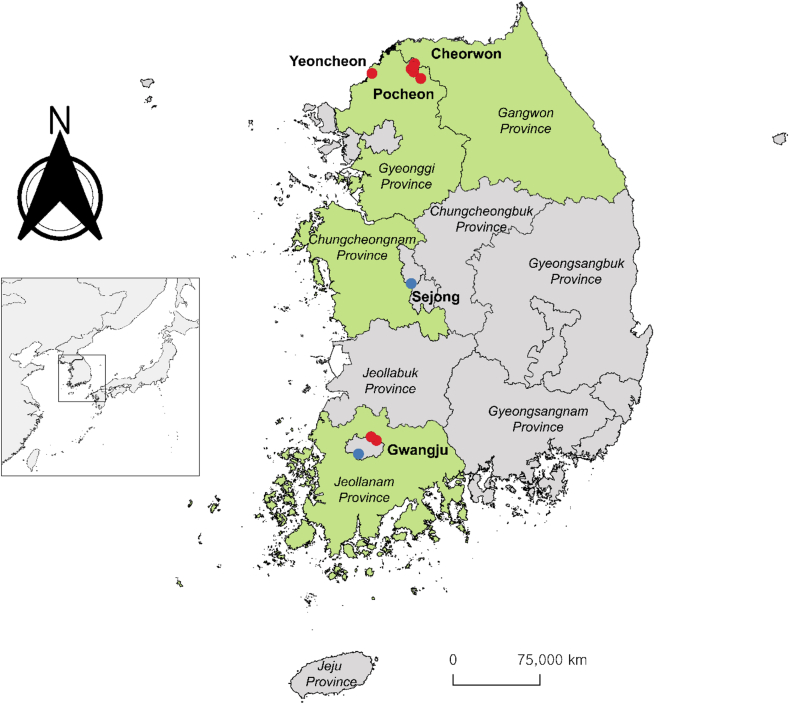
The map shows the trapping locations for collecting *C. lasiura* in 2023. From 24 shrew samples, three samples detected positive for Langya virus Korea (LayV KOR) and twelve samples detected positive for Gamak virus (GAKV). The green-colored provinces indicate areas covered in the 2023 shrew surveillance project. The red-colored dots represent areas with paramyxovirus-positive samples, while the blue-colored dots represent areas with paramyxovirus-negative samples. (For interpretation of the references to colour in this figure legend, the reader is referred to the web version of this article.)

In 2023, we collected kidney tissues from 24*C. lasiura* from the Gyeonggi, Gangwon, Chungcheongnam, and Jeollanam Provinces (Fig. 1). The animal handling was performed according to the Korea University Institutional Animal Care and Use Committee (#2022–34). The specimens were screened for paramyxoviruses using degenerate primer and the reverse transcription PCR method described previously [6,7]. We identified the 400 to 500 bp partial sequences using BLASTn, aligned them using MUSCLE 5, and conducted phylogenetic analysis using IQTREE and the Interactive Tree of Life (ITOL) [[8], [9], [10]].

Metagenomic raw reads were obtained from our previous study on the kidney samples of the shrew species in 2017 [5]. The raw reads were re-analyzed using de novo assembly, followed by mapping to a reference to verify the complete virus genome. The QIAGEN CLC Genomic Workbench (https://digitalinsights.qiagen.com) was used for the bioinformatic analysis. The 3′ leader and 5′ trailer sequences were determined using Rapid Amplification of cDNA Ends (RACE) PCR with the SMARTer RACE 5′/3′ (TaKaRa Bio Inc., Japan).

The complete virus sequences served as the template for designing amplicon-based sequencing primers using PrimalScheme [11]. The cDNAs from the 2023*C. lasiura* paramyxovirus-positive samples were used as a template for tiled PCR following the ARTIC Network protocol (https://artic.network/2-protocols.html). Amplicon-based sequencing was performed on a MinION platform with R10 flow cell chemistry and the Native Barcoding Kit 24 V14 (Oxford Nanopore Technologies, UK). Raw reads were demultiplexed, trimmed, and mapped to the complete virus genome obtained from the previous analysis using the QIAGEN CLC Genomic Workbench toolkit. The consensus sequence was annotated based on the parahenipaviruses from the NCBI database.

## 3 Result and discussion

Detailed sample characteristics are listed in Table 1. The positivity rate for paramyxovirus was 62.5 %, with a higher rate in males than in females (76.9 % vs. 45.5 %). The captured animals exhibited a high positivity rate in the adult group (75 %). Geographically, we obtained more samples from the northern than the southern areas, with the highest positivity rate (71.4 %) in Gyeonggi Province. Notably, 3 of 15 (20 %) partial sequences from the ROK shared a common ancestor with LayV from China (Supplementary Fig. 1).

**Table 1:** Prevalence of *Parahenipavirus* in *Crocidura lasiura* samples collected in the ROK in 2023.

Categories		Positive for *Parahenipavirus* (%)			Negative for *Parahenipavirus* (%)	Total^c^ (%)
		Total^a^	LayV^b^	GAKV^b^		
Sex	Male	10 (76.9)	3 (30)	7 (70)	3 (23.1)	13 (54.2)
	Female	5 (45.5)	–	5 (100)	6 (55.5)	11 (45.8)
Age^d^	Sub-adult	–	–	–	4 (100.0)	4 (16.7)
	Adult	15 (75.0)	3 (20)	12 (80)	5 (25.0)	20 (83.3)
Province	Gyeonggi	10 (71.4)	3 (10)	7 (70)	4 (28.6)	14 (58.3)
	Gangwon	1 (50.0)	–	1 (100)	1 (50.0)	2 (8.3)
	Chungcheongnam	–	–	–	2 (100.0)	2 (8.3)
	Jeollanam	4 (66.7)	–	4 (100)	2 (33.3)	6 (25.1)
**Total**		**15 (62.5)**	**3 (20)**	**12 (80)**	**9 (37.5)**	**24 (100)**

a , The percentage is based on the total for each row.

b , The percentage is based on the total number of positive samples for each category.

c , The percentage is based on a total sample size of 24.

d , The shrew age grouping is <10 g for sub-adult and ≥10 g for adult.

**Fig. 2:**
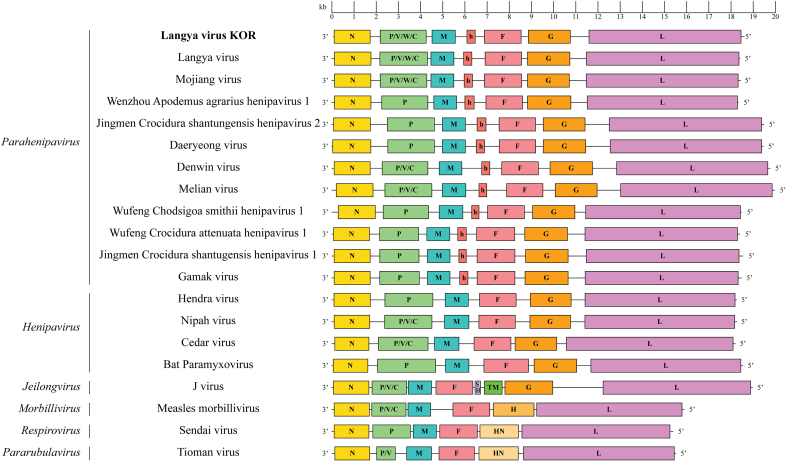
The genome organization of Langya virus Korea (LayV KOR) compared with the LayV from China and other reported Parahenipaviruses. Abbreviations: N, nucleocapsid protein; P, phosphoprotein; M, matrix protein; h, hypothetical protein; F, fusion protein; SH, small hydrophobic protein; TM, transmembrane protein; G, glycoprotein; H, hemagglutinin protein; HN, hemagglutinin-neuraminidase protein; L, large protein.

We retrospectively analyzed the metagenomic data from our previous study in 2017, which led to the discovery of GAKV in *C. lasiura* [5]*.* We focused on Cl17–6 and Cl17–9, whose partial sequences clustered phylogenetically independent of GAKV. This approach resulted in long contigs exhibiting 79.7 % and 79.8 % nucleotide similarity to LayV China, which were subsequently designated as LayV KOR Cl17–6 and Cl17–9. Annotation of the open reading frames (ORFs) from 3′ to 5′ (Fig. 2) showed the genomic organization as nucleocapsid (N), phosphoprotein (P), matrix protein (M), hypothetical protein (h), fusion protein (F), glycoprotein (G), and large protein (L). The complete genome sequence of LayV KOR was 18,420 nucleotides (nt) in length, with the leader sequence ACCAAA at the 3′ end and the trailer sequence TGTGGT at the 5′ end. The sequential amplicon-based sequencing of the 2023 paramyxovirus-positive samples generated three complete sequences, designated as LayV KOR Cl23–2, Cl23–18, and Cl23–19, respectively.

We obtained complete genomes that fulfilled the rule of six, a requisite for productive paramyxovirus genomes. The full genome sequence length was 18 nt longer than that of LayV from China, with differences in the non-coding terminals. Our analysis identified a 366 nt ORF, designated as a “hypothetical protein” (h) between the M and F genes. The hypothetical protein was found in other *Parahenipavirus* including Denwin virus and Wufeng *Chodsigoa smithii* henipavirus 1. This ORF was only observed from *Parahenipavirus* originated in shrews and rodents. Notably, the canonical gene start signal for the hypothetical protein was located after the M gene stop signal and intergenic region (IGR), and a non-canonical stop-IGR-start signal was identified between the h- and F-coding sequences. The biological significance of this hypothetical protein remains to be investigated.

**Fig. 3:**
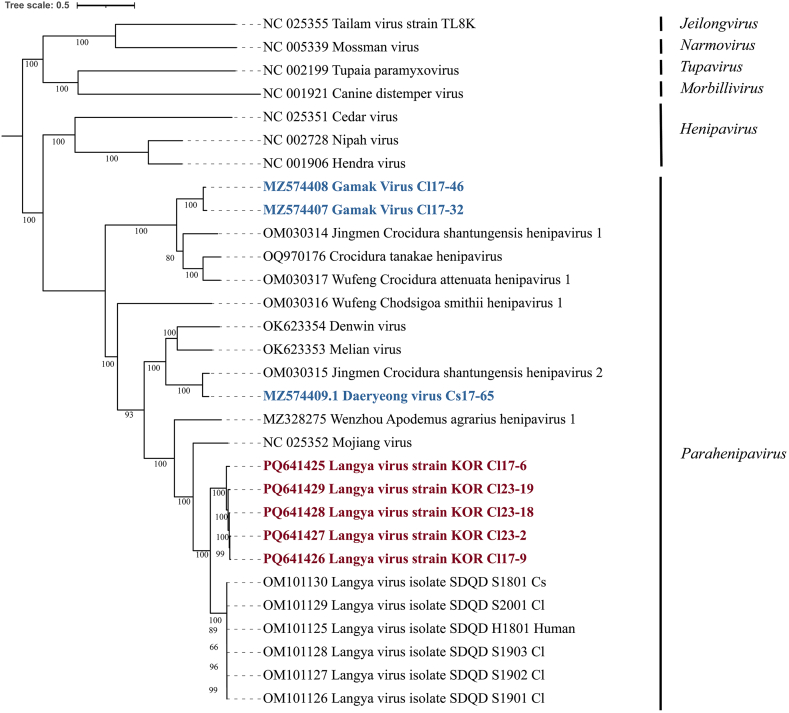
Phylogenetic tree of the Langya virus Korea (LayV KOR) complete sequences aligned with other paramyxoviruses. The tree was constructed using maximum likelihood analysis via IQTREE web server with the GTR + F + I + G4 model selected based on BIC and 1000 bootstrapping. LayV strains KOR Cl17–6 and Cl17–9 were obtained through reanalysis of metagenomic data from 2017, while Cl23–2, Cl23–18, and Cl23–19 were obtained through amplicon-based sequencing of the 2023 surveillance samples. The burgundy-colored labels represent the LayV KOR reported in this study, while the blue-colored labels represent the *Parahenipavirus* previously found in the Republic of Korea. (For interpretation of the references to colour in this figure legend, the reader is referred to the web version of this article.)

**Fig. 4:**
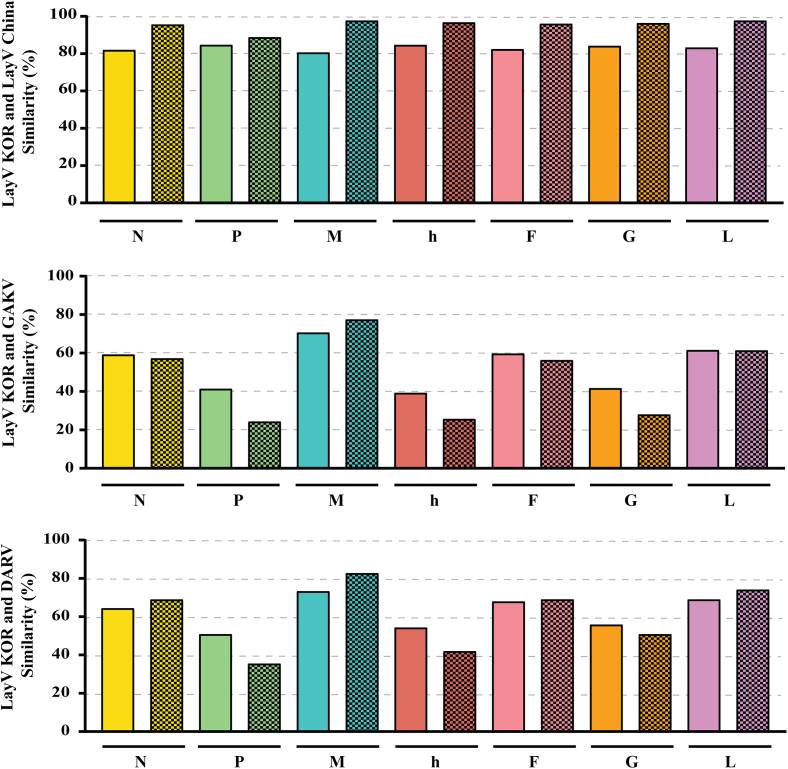
Percentage similarity of the genomic and amino acid sequences of the Langya virus Korea (LayV KOR) coding sequence against LayV China, Gamak virus (GAKV), and Daeryeong virus (DARV). The bar with a solid colour represents the homology of nucleotide sequences. The bar with a checkered pattern indicates the homology of amino acid sequences. Abbreviations: N, nucleocapsid protein; P, phosphoprotein; M, matrix protein; h, hypothetical protein; F, fusion protein; G, glycoprotein; L, large protein.

The phylogenetic analysis of five complete LayV KOR genome sequences revealed a common ancestor with LayV China but exhibited a distinct genetic lineage (Fig. 3). The diversification between LayV KOR and LayV China may have resulted from geographical influences. Similarity analysis of the coding sequences (CDSs) and their translated protein sequences compared to LayV China demonstrated average similarities of 83.0 % and 95.5 %, respectively. In contrast, nucleotide similarity dropped to an average of 53.3 % and 62.2 % when compared with the CDSs of GAKV and DARV. At the amino acid level, the similarity of these Parahenipaviruses to LayV KOR was even lower, ranging from 47.1 % for GAKV to 60.4 % for DARV (Fig. 4). The phylogenetic analysis and high sequence similarity between LayV KOR and LayV China suggest a closer evolutionary relationship compared to other Parahenipaviruses found in the ROK. The significant amino acid similarity between LayV KOR and LayV China also indicates that LayV KOR might elicit a similar immune response. However, the disease mechanisms of LayV are yet to be elucidated.

LayV China was first identified in clinical samples collected in 2018, whereas our group observed LayV KOR from shrew samples collected in 2017 and 2023. To date, no human-to-human transmission or deaths associated with LayV infection have been reported. The current number of clinical cases remains low, limiting the ability to assess the risk to vulnerable groups. LayV infection lacks pathognomonic signs, with fever being its primary symptom. The index case of LayV was detected through active surveillance using metagenomic sequencing in patients with fever and a history of animal exposure [2]. This led to a hypothesis that LayV KOR infections in the Korean population might have been underdiagnosed since the sentinel monitoring is infrequent. Initial LayV cases were predominantly found in farmers (85 %), suggesting that outdoor activities might serve as a potential route for human exposure to shrews. In humans, LayV was detected using throat swabs, indicating viral shedding in the respiratory tract. Similar to other respiratory viruses of zoonotic origin, including betacoronaviruses and influenza viruses, LayV has the potential for rapid transmission if it further adapts to infect humans [12,13].

This study has several limitations: the current absence of viral isolates, the limited geographic surveillance and the unexplored potential for human exposure. The tropism of LayV KOR for shrews may hinder effective virus isolation using common cell lines, which originate from rodent or simian genera. Therefore, the development of shrew cell lines or transgenic cell lines to generate an appropriate receptor is necessary.

## 4 Conclusion

Our findings provide evidence for the circulation of LayV in *C. lasiura* in the ROK, which closely related to a zoonotic pathogen previously identified exclusively in China. This report raises awareness and caution among physicians regarding clinical cases of emerging *Parahenipavirus*. Future studies should assess evidence of LayV KOR exposure in humans by developing antibody screening for active serosurveillance in the ROK.

## CRediT authorship contribution statement

**Augustine Natasha:** Writing – review & editing, Writing – original draft, Methodology, Investigation, Formal analysis, Data curation, Conceptualization. **Sarah E. Pye:** Writing – review & editing, Validation, Formal analysis. **Kyungmin Park:** Writing – review & editing, Validation, Project administration, Methodology, Formal analysis, Data curation. **Shivani Rajoriya:** Methodology, Formal analysis. **Intae Yang:** Visualization, Formal analysis. **Jieun Park:** Writing – review & editing, Visualization. **Haryo Seno Pangestu:** Visualization, Methodology. **Jongwoo Kim:** Methodology, Data curation. **Yeonsu Oh:** Writing – review & editing, Funding acquisition. **Carolina B. López:** Writing – review & editing, Funding acquisition, Conceptualization. **Jin-Won Song:** Writing – review & editing, Funding acquisition. **Won-Keun Kim:** Writing – review & editing, Supervision, Investigation, Funding acquisition, Conceptualization.

## Funding

This research was supported by the 10.13039/501100011705Korea Institute of Marine Science & Technology Promotion (KIMST) funded by the 10.13039/501100003566Ministry of Oceans and Fisheries, Korea (RS-2021-KS211475), a 10.13039/501100003653Korea National Institute of Health research project (2024-ER2502–00), and Government-wide R&D to Advance Infectious Disease Prevention and Control, Republic of Korea (RS-2023-KH140418). This work was supported by the NRF Korea grant funded by the Korean government (MSIT) (2023R1A2C2006105). This research was also supported by a 10.13039/501100009708Novo Nordisk Foundation PAD award (NF22SA0082041) and BJC investigator funds. This study was partly supported by the NIH grant (U01 AI151810).

## Declaration of competing interest

The authors declare no competing financial interests.

## Data Availability

All the sequences are available in the NCBI GenBank database with accession number PQ641425-PQ641429.

## Appendix A Supplementary data

Supplementary figure phylogenetic tree partial sequences Image 1
