# Supplementary material for: Detection and characterization of Langya virus in Crocidura lasiura (the Ussuri white-toothed shrew), Republic of Korea
Source: One Health. 2025 Mar 19;20:101017. doi: 10.1016/j.onehlt.2025.101017 (PMC11982054; doi:10.1016/j.onehlt.2025.101017)
Supplement: Supplementary file 1 — Supplementary figure phylogenetic tree partial sequences [file mmc1.docx]

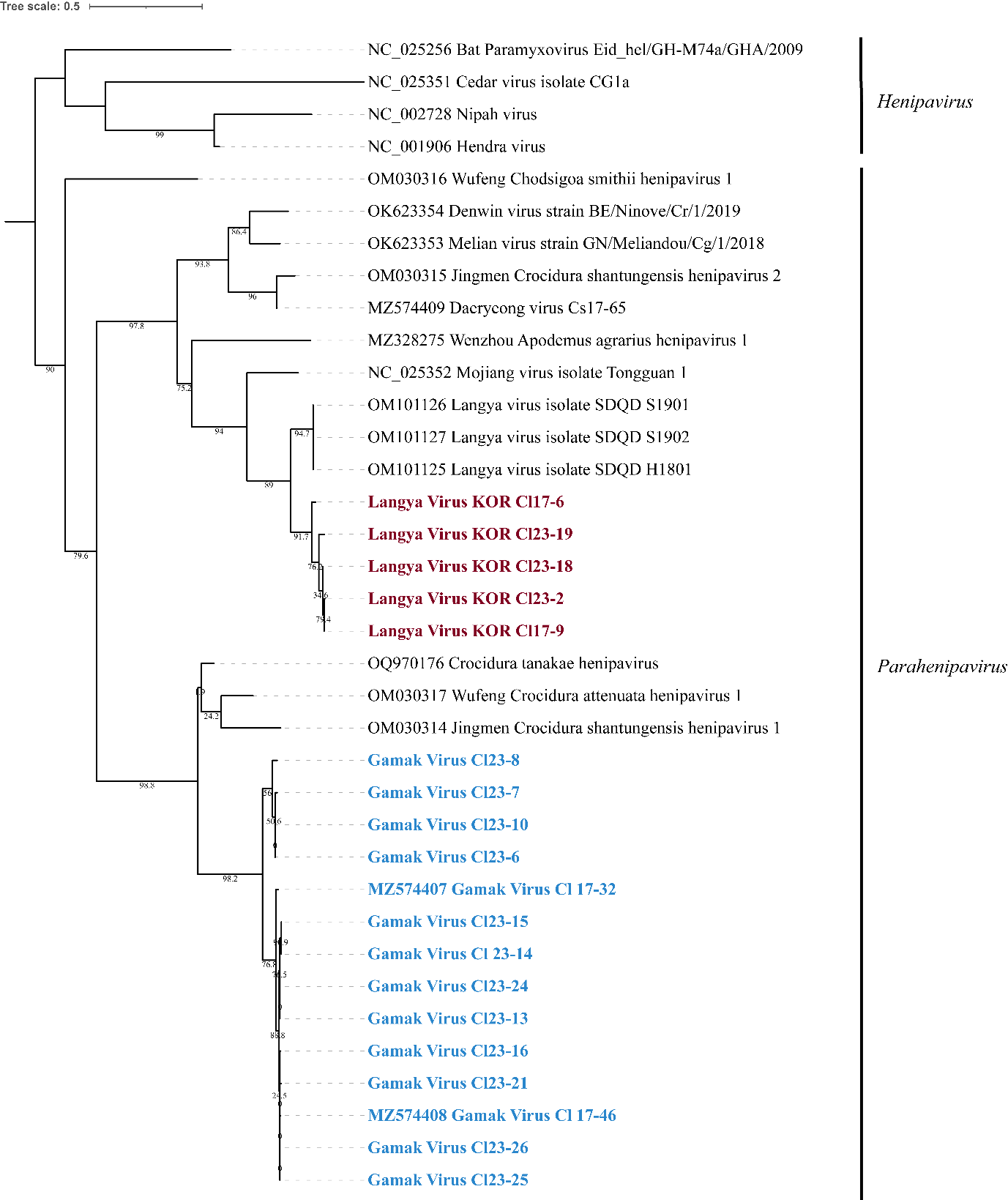


Supplementary figure 1. The phylogenetic tree of the collected partial sequences from paramyxovirus PCR screening. The Langya Virus KOR Cl17-6 and Cl17-9 were the partial sequence from our previous study in 2017, while the Cl23-2, Cl23-18, and 23-19 were the partial sequence from shrew samples collected in 2023. The tree was constructed using maximum likelihood analysis by IQTREE web server, with GTR+F+I+G4 model chosen according to BIC and 1000 bootstrapping. Burgundy-colored labels represent samples with partial sequences identified as Langya Virus KOR, while the blue colored labels represent partial sequences identified as Gamak Virus.
